# Effects of Di(2-ethylhexyl) Phthalate (DEHP) on Female Fertility and Adipogenesis in C3H/N Mice

**DOI:** 10.1289/ehp.1104016

**Published:** 2012-05-15

**Authors:** Juliane-Susanne Schmidt, Kristina Schaedlich, Nadia Fiandanese, Paola Pocar, Bernd Fischer

**Affiliations:** 1 Department of Anatomy and Cell Biology, Martin Luther University Faculty of Medicine, Halle (Saale), Germany; 2 Dipartimento di Patologia Animale, Igiene e Sanità Pubblica Veterinaria, Facoltà di Medicina Veterinaria, Università degli Studi di Milano, Milan, Italy

**Keywords:** adiponectin, adipose tissue, DEHP, endocrine disruptors, female reproduction, leptin, obesity, phthalates

## Abstract

Background: Di(2-ethylhexyl) phthalate (DEHP) and its metabolites are known to affect lipid metabolism and adipogenesis, mainly by activation of peroxisome proliferator-activated receptors (PPARs). Exposure to DEHP has been linked with testicular impairment and male subfertility. However, the effects of DEHP on female reproductive health and metabolism have not been studied in detail.

Objective: We examined the effects of dietary DEHP exposure on metabolism and fertility in female mice.

Methods: In two independent approaches, female C3H/N mice were exposed to DEHP (0.05, 5, or 500 mg/kg of body weight per day) via their diet for 8 weeks, and we recorded food intake, weight gain, and litter size. After exposure, liver, visceral fat, and plasma from F0 females (study I) and F0 dams and their F1 offspring (study II) were analyzed by quantitative real-time polymerase chain reaction and enzyme-linked immunosorbent assay.

Results: In study I, DEHP-exposed F0 females (all dose groups) had a significant increase in body weight, food intake, and visceral adipose tissue compared with controls. In the 500-mg DEHP group, PPARα and PPARγ transcripts were significantly changed in liver tissue. In the same group, *PPAR*γ mRNA was significantly reduced in liver but not in fat tissue. In addition, leptin and *FABP4* (fatty acid binding protein 4) mRNA were increased in adipose tissue, whereas adiponectin was decreased. In study II, we detected a 100% abortion rate in F0 dams in the 500-mg group. F1 offspring exposed *in utero* and during lactation had an increase in visceral fat tissue and body weight.

Conclusion: Fertility was impaired in mice exposed to high doses of DEHP, and body weight and visceral fat deposits were increased in mice exposed to environmentally relevant doses. Although F1 mice were exposed to DEHP only *in utero* and during lactation, we observed metabolic changes in the offspring of diet-exposed females.

Endocrine-disrupting chemicals (EDCs) have been hypothesized to contribute to the high prevalence of diseases such as obesity, hypertension, and diabetes mellitus (Grün and Blumberg 2009; Heindel and vom Saal 2009; Latini et al. 2010). EDC exposure occurs during all phases of life. Early EDC exposure in the developing embryo may cause permanent metabolic alterations (Fowler et al. 2012), thus potentially contributing to development of obesity later in life.

One EDCs is the plasticizer di(2-ethylhexyl) phthalate (DEHP); it is commonly used in a wide range of products including food packages, cosmetics, medical devices, and polyvinyl chloride (PVC) (Hauser and Calafat 2005). In Western Europe, DEHP accounts for about 18% of all plasticizers used (European Union 2008). Because of its potential adverse effects on human health, the European Union banned the use of DEHP in children’s products in 2004 [Directive 2005/84/EC (European Union 2005)].

Humans are exposed to DEHP through ingestion, inhalation, and dermal exposure for their lifetimes, including intrauterine life. Although maternal DEHP exposure has been associated with impaired gonadal development and fertility in human males (Hauser et al. 2007; Pant et al. 2008; Sharpe 2006; Swan 2008), its effects on female reproductive health and the developing embryo have not been studied in detail. Previous studies have detected DEHP in serum and urine samples of pregnant women, cord blood collected at birth (Adibi et al. 2003; Latini et al. 2003), and in breast milk (Frederiksen et al. 2007; Main et al. 2005).

Besides being a potential risk for reproductive health, possibly in females as well as in males, DEHP and its metabolite mono(2-ethylhexyl) phthalate exert metabolic effects by activation of peroxisome proliferator-activated receptors (PPARs) α and γ, key mediators of lipid metabolism and adipogenesis (Feige et al. 2007; Lapinskas et al. 2005). Because of their high prevalence, obesity and the related disorder dyslipidemia have become global health risks (Ogden et al. 2007) and are sometimes termed an “obesity pandemic.” In addition, dyslipidemia is associated with both obesity and subfertility (Carr and Brunzell 2004; Pasquali and Gambineri 2006). By 1995, about 200 million people worldwide exhibited obesity and, in 2000 this number increased to 300 million [World Health Organization (WHO) 2000]. Actually, overweight affects 30–70% of adults in the WHO European Region and up to 60% of children before puberty (WHO 2012). The multifactorial reasons for the obesity pandemic are being intensively studied but are not yet understood. Stahlhut et al. (2007) found a positive and significant correlation between the concentrations of several phthalate metabolites and abdominal obesity in adult U.S. males based on data from the National Health and Nutrition Examination Survey (NHANES) 1999–2002. However, surprisingly little is known about how DEHP might interfere with female health and obesity (Davis et al. 1994; Lamb et al. 1987; Stahlhut et al. 2007; Takai et al. 2009).

In the present study, we investigated the effects of DEHP on fertility and growth of exposed mice and their offspring. We chose concentration ranges ranging from “real world” exposure levels—mirroring the mean daily intake by the normal population and the NOAEL (no observed adverse effect level) of DEHP [0.05 and 5 mg/kg of body weight (bw)/day] (Kavlock et al. 2002)—up to high DEHP levels (500 mg/kg bw/day). We studied general health parameters and analyzed the expression patterns of key genes involved in lipid metabolism.

## Materials and Methods

*Animal care, diets, and DEHP exposure.* The experimental protocol was approved by the Animal Use and Care Committee (Regierungspräsidium Sachsen-Anhalt, Dessau, Germany). All experimental animals were treated humanely and with regard for alleviation of suffering. Mice were housed individually under controlled light and temperature conditions (12 hr light/dark cycle; 22 ± 1°C) with free access to food and water.

We randomly divided mature female C3H/N mice (purchased from Charles River, Sulzfeld, Germany) weighing 18–23 g into four groups and exposed them to different concentrations of dietary DEHP (0, 0.05, 5, and 500 mg/kg bw/day) for a period of 8 weeks. Food intake and weight gain were measured twice weekly.

For the production of DEHP-containing diets, DEHP (Sigma-Aldrich, Taufkirchen, Germany) was diluted in commercial sunflower oil (Maggi GmbH, Frankfurt/Main, Germany) and applied to rodent chow (Altromin, Lage, Germany). All DEHP containing diets were produced with the end volume of 60 g oil/1 kg diet preparation. The control diet contained the same amount of sunflower oil vehicle. Because of the same composition of the diets, experimental and control diets had the same caloric density. The DEHP concentration range (0.05, 5, and 500 mg/kg bw/day] included dose levels relevant to human exposure (Kavlock et al. 2002), the NOAEL, and high doses known for adverse reproductive and developmental effects in animal studies (Moore et al. 2001). The DEHP concentrations in animal diets were verified by an accredited laboratory (SGS GmbH Germany, Hamburg, Germany) before being used in the present study. For the DEHP-containing diets, actual DEHP ingestion had to be calculated to ensure that changes in food intake did not change the estimated DEHP intake. In a pilot study, we measured daily food intake in female C3H/N mice. Using these data we estimated the daily DEHP intake to be 0.00102 mg, 0.108 mg, and 10.60 mg DEHP for the 0.05-, 5-, and 500-mg/kg bw/day groups, respectively.

*Effects of DEHP on F0 females (study I).* In three independent experiments (two with 10 animals/group and one with 5 animals/group), females received the diets for 8 weeks, and body weights were recorded weekly. In the last week of exposure, female F0 mice were superovulated by intraperitoneal (ip) injection of 7.0 IU pregnant mare’s serum gonadotropin (PMSG), injected 48 hr later with 7.5 IU human chorionic gonadotropin (hCG; Calbiochem, Darmstadt, Germany), and mated with unexposed males. The vaginal plug was checked the next morning, and females were sacrificed by cervical dislocation 92 hr after hCG injection. We collected F1 embryos by flushing the excised uteri with 0.2 mL phosphate-buffered saline (PBS) and 4% polyvinyl alcohol. The morphology of embryos was assessed by light microscopy (KL 1500 LCD; Carl Zeiss, Oberkochen, Germany). Embryos with fragmented or disaggregated blastomeres were categorized as degenerated embryos. Preserved tissues (liver and visceral fat) were immediately frozen in liquid nitrogen and stored at –80°C until use. We collected blood specimens by heart puncture using Monovettes (Sarstedt, Nümbrecht, Germany), centrifuged blood samples (3,500 × *g* for 5 min), and stored the plasma at –20**°**C prior to analysis. We determined the plasma concentrations of leptin by enzyme-linked immunosorbent assay (ELISA; ChrystalChem, Downers Grove, IL, USA).

*Effects of DEHP on F0 dams and* in utero/*lactationally exposed F1 offspring (study II).* In two independent experiments (one with 10/animals/group and one with 5 animals/group), female mice were exposed to 0, 0.05, 5, or 500 mg DEHP/kg/day for 8 weeks. After the first week of feeding, the females were mated with unexposed males and the vaginal plug was checked. The times to pregnancy between dams ranged from 2 to 7 days. The time intervals between exposure and mating were the same for DEHP-exposed and control animals. The mice were weighed weekly and allowed to give birth. We detected spontaneous abortion by bloody residues and weight loss of dams. At weaning, dams were sacrificed by cervical dislocation.

F1 offspring were exposed to DEHP only via placenta and breast milk until weaning. At weaning on postnatal day (PND) 21, we sexed all pups and recorded their body weights. The mean body weight was calculated for at least 12 litters/treatment group (control: 25 females, 22 males; 0.05 mg DEHP: 28 females, 27 males; 5 mg DEHP: 22 females, 23 males). For intergenerational analysis, we randomly seleced 12 F1 males and 12 F1 females per treatment group on PND21; these mice received standard chow without DEHP (Altromin). On PND84 we recorded their body weights. Sexually mature F1 females (PND84) were stimulated by ip injection of 7.0 IU PMSG, injected 48 hr later with 7.5 IU hCG, and mated with unexposed males. Ninety-two hours after hCG administration, F1 females were sacrificed by cervical dislocation and tissues were collected as described for study I.

*Reagents for molecular biology.* We purchased the Superscript II Reverse Transcriptase (RT) kit, deoxyribonucleotide triphosphate (dNTPs), and Taq polymerase from Invitrogen (Karlsruhe, Germany); restriction enzymes from New England Biolabs (Frankfurt, Germany); random primers from Roche (Mannheim, Germany); and RNAse inhibitor from Promega (Mannheim, Germany).

*RNA extraction and RT reaction.* We extracted total RNA from liver and visceral fat tissue as described previously by Chomczynski and Sacchi (1987). RNA was treated with DNAse for 1 hr. The amount of total RNA was determined spectrophotometrically at 260 nm and RNA purity was determined by the 230/280 nm ratio. RNA was used only if the 260/280 nm ratio was > 1.8. Three micrograms of total RNA were reverse transcribed in a volume of 20 μL containing 10 mM dNTPs, 0.1 M dithiothereitol, 200 units superscript II, 20 units RNAse inhibitor, 1 μL random primer, and 2 μL RT buffer at 42°C for 1 hr, followed by incubation at 70°C for 10 min. As a control for DNA contamination, 2 μg RNA was amplified by polymerase chain reaction (PCR) without the RT reaction. This control reaction was performed for each primer combination and in all PCR amplifications. Resulting PCR products were separated by electrophoresis. To confirm the amplification product, the resulting RT-PCR products were visualized in a 2.0% agarose gel and sequenced.

*Quantitative real-time PCR (qRT-PCR).* We analyzed samples by qRT-PCR using an iQ5 Optical System (Bio-Rad Laboratories, Herts, UK) and SYBR green master mix (Eurogentec, Köln, Germany), a double-stranded DNA–specific fluorescent dye. Primer sequences are listed in Table 1. Each assay included duplicates of each cDNA sample and a “no-template” control. The parameter cycle threshold (CT) is defined as the cycle number at which fluorescence intensity exceeds a fixed threshold. Absolute mRNA expression was calculated by means of specific plasmid standards, using serial dilutions (10^8^, 10^7^, 10^6^, 10^5^). The expression of the housekeeping gene *18S* rRNA was used to normalize samples for the amount of cDNA used per reaction. To confirm the amplification, the resulting qRT-PCR products were analyzed by dissociation curves and visualized in an agarose gel.

**Table 1:** Primers used for real time RT-PCR.

Gene	Sequence 5’–3’	Sequence 3’–5’	Base pairs
PPARα		TCTCCCCATTTCTCATCCTG		GCCAGGACTGAAGTTCAAGG		170
PPARγ		GATGGAAGACCACTCGCATT		AACCATTGGGTCAGCTCTTG		115
Leptin		ATCTATGTGCACCTGAGGGTAGA		TCCTTTTCACAAAGCCACACTAT		151
Adiponectin		TGTTGGAATGACAGGAGCTG		CGAATGGGTACATTGGGAAC		147
FABP4		TCGACTTTCCATCCCACTTC		TGGAAGCTTGTCTCCAGTGA		282
18S		AGAAACGGCTACCACATCCAA		CCTGTATTGTTATTTTTCGTCACTACCT		91
FABP4, fatty acid protein 4.						

*Histological examination of visceral fat tissue.* Visceral fat tissue from control and DEHP-exposed mice was fixed in PBS containing 4% paraformaldehyde (pH 7.5) for 24 hr, embedded in paraffin, sectioned (6 µm), and stained with hematoxylin/eosin. The slides were examined by light microscopy (BZ-8100; Keyence Germany, Frankfurt, Germany); we analyzed three fields in three histological sections from each animal.

*Statistical analysis.* Statistical differences between groups in study I (except for body weight gain) and study II were tested using one-way analysis of variance (ANOVA) followed by Duncan’s post hoc test using SigmaPlot, version 11.0 (Systat Software GmbH, Erkrath, Germany). Differences between groups were considered statistically significant if *p* < 0.05. Data are presented as mean ± SE. Data for body weight gain (study I) were analyzed by a generalized linear model (GLM; SPSS Statistics 17; IBM Deutschland GmbH, Ehningen, Germany) using the DEHP feeding period, experimental replicate, and DEHP treatment group as factors. Differences in body weight gain of DEHP-exposed groups compared with the control group during the DEHP feeding period were adjusted to the experimental replicate. We used the Greenhouse–Geisser correction to correct for violations of sphericity, which is the extension of the variance homogeneity assumption needed for ANOVA in the case of repeated measurement ANOVA. Controls were used as the reference group.

## Results

**Figure 1:**
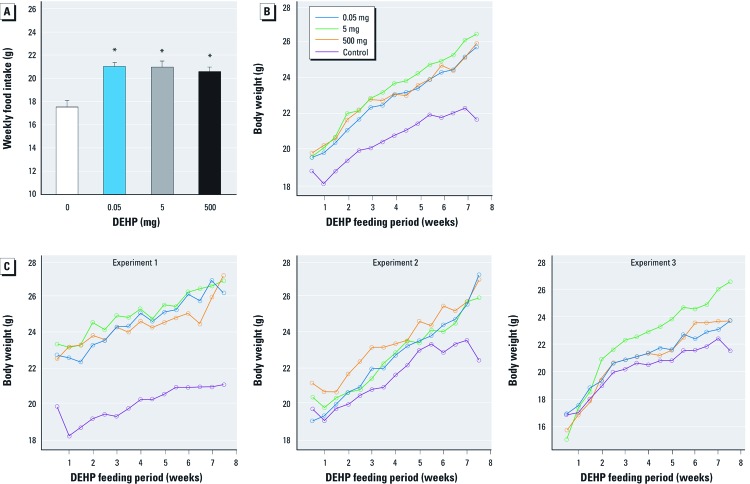
Effects of 8 weeks of dietary exposure to DEHP on food intake and body weight in female C3H/N F0 mice: study I. (*A*) Mean weekly food intake (± SE). (*B*) Body weight gain from experiments 1–3 combined (25 animals/treatment group). (*C*) Body weight gain from individual experiments 1, 2, and 3 (*C*); in experiments 1 and 3, *n* = 10 animals/treatment group, and in experiment 2, *n* = 5 animals/treatment group. *p < 0.05 compared to controls.

*Study I.* Weekly food intake and body weight. Daily exposure to dietary DEHP for 8 weeks did not adversely affect general health; we observed no clinical signs of toxicity in F0 females. Weekly food intake was up to 20% higher in all DEHP-exposed groups compared with controls (*p* < 0.05) (Figure 1A). At the end of the 8 weeks, the body weights of DEHP-exposed F0 mice (all three dose groups) were significantly higher than those of controls: 0.05 mg DEHP, 2.15 g [95% confidence interval (CI): 1.47, 2.82]; 5 mg DEHP, 2.77 g (95% CI: 2.09, 3.44); 500 mg DEHP, 2.36 g (95% CI: 1.68, 3.03); all *p* < 0.001] (Figure 1B). Although significant variations occurred between individual experiments within treatment groups (*p* < 0.001), body weight was increased in DEHP-exposed mice in each experiment (Figure 1C).

**Figure 2:**
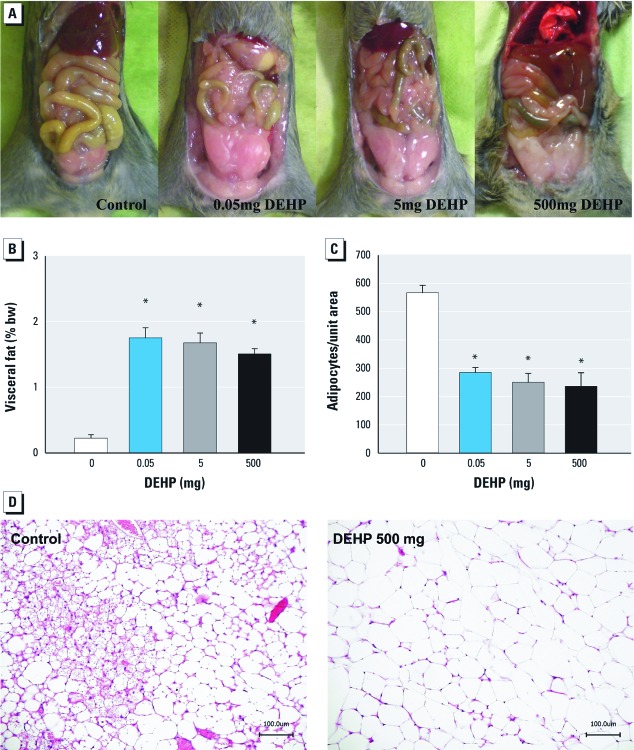
Effects of 8 weeks of dietary exposure to DEHP on visceral fat in female C3H/N F0 mice: study I. (*A*) Visceral fat in mice. (*B*) Amount of visceral fat tissue presented as a percentage of body weight. (*C*) Number of adipocytes counted per unit area. (*D*) Representative histological sections of adipose tissue; bar = 100 µm. In *B* and *C*, results are mean ± SE from three independent experiments; *n* = 25 animals/treatment group. *p < 0.05 compared to controls.

Visceral fat tissue. After the 8-week DEHP exposure period, DEHP-treated F0 mice had more visceral fat tissue than controls (Figure 2A). This increase was not dose-dependent (Figure 2B). To better characterize this increase, we examined histological sections of visceral adipose tissue in a double blind test; we found that the adipocytes of DEHP-exposed mice were larger (hypertrophied) than those of controls (Figure 2C,D).

**Figure 3:**
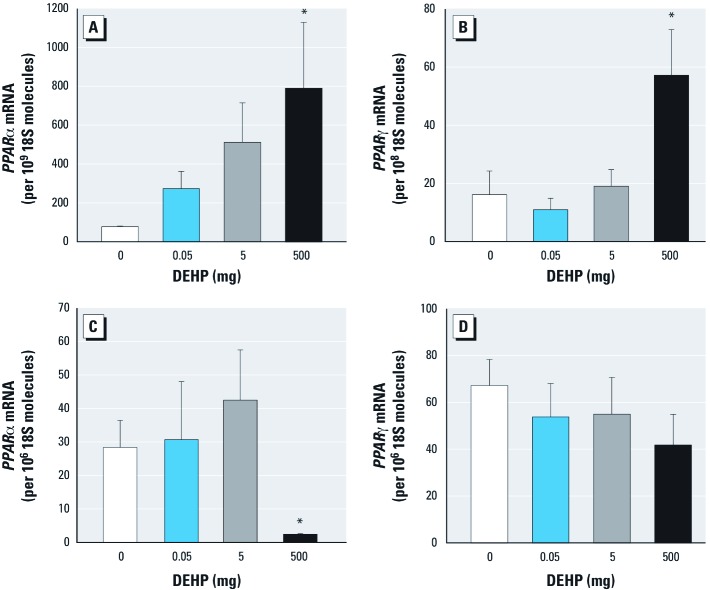
Effects of DEHP dietary exposure on expression of *PPAR* isoforms in female C3H/N F0 mice: study I. Absolute mRNA amounts of *PPAR*α and *PPAR*γ in liver (*A*,*B*) and visceral fat tissue (*C*,*D*). Results are mean ± SE from three independent experiments (*n* = 5 animals/ treatment group). *p < 0.05 compared to controls.

Expression of PPAR isoforms in liver and visceral fat tissue of F0 mice. We analyzed mRNA expression of PPARα transcripts/10^9^ 18S molecules (Figure 3A) and PPARγ transcripts/10^8^ 18S molecules in the liver. The increase in *PPAR*α and *PPAR*γ mRNA expression in the 500-mg DEHP group was significant (*p* < 0.05). mRNA expression of PPARα transcripts/10^6^ 18S molecules in visceral fat tissue was significantly (*p* < 0.05) decreased in the highest treatment group (Figure 3C), whereas PPARγ transcripts were comparable among all groups (Figure 3D).

**Figure 4:**
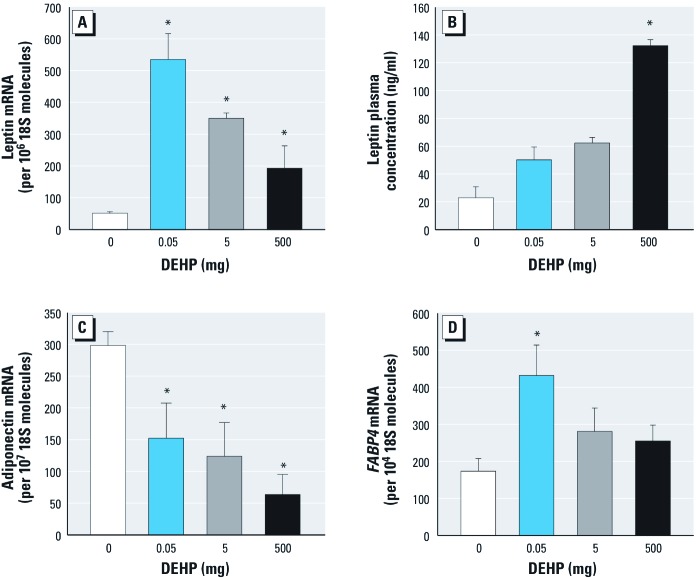
Effects of DEHP dietary exposure on mRNA concentrations in visceral fat tissue: study I. (*A*) Absolute mRNA concentration of leptin. (*B–D*) Plasma leptin mRNA concentrations (*B*) and absolute mRNA concentrations of adiponectin (*C*) and fatty acid binding protein 4 (*FABP4*; *D*). Results are mean ± SE from three independent experiments (*n* = 5 animals/group). *p < 0.05 compared with controls.

Plasma concentration and expression of adipokines in visceral fat tissue. DEHP treatment was associated with significant increases in leptin mRNA transcripts/10^6^ 18S molecules in adipose tissue, with the highest elevation in the lowest dose group Figure 4A). The plasma concentration of leptin was significantly higher in the 500-mg DEHP treatment group compared with controls (Figure 4B). Adiponectin mRNA levels were significantly decreased in all DEHP treatment groups (Figure 4C).

*FABP4* (fatty acid protein 4) mRNA levels in visceral fat tissue. The mRNA concentration of *FABP4*, a specific carrier protein for fatty acids in adipose tissue, was significantly increased in mice exposed to 0.05 mg DEHP compared with controls (Figure 4D). In the higher treatment groups, *FABP4* expression/10^4^ 18S molecules was only slightly higher than in controls.

F1 preimplantation embryos. We observed no significant differences in the average number of flushed embryos per mouse at 4 days post coitum (dpc) between treatment groups. However, DEHP exposure was associated with a nonsignificant increase in the incidence of degenerated blastocysts in the highest DEHP dose group (control, 14%; 500 mg DEHP, 32%) (Table 2).

**Table 2:** Pregnancy rate and F1 embryo survival in superovulated F0 female mice: study I.

DEHP (mg)						
0	0.05	5	500
Total dams (n)		25		25		25		25
Pregnant dams (n)		19		21		23		17
Flushed embryos/dam (mean ± SE)		8 ± 0.2		9 ± 1.2		9 ± 1.2		7 ± 1.4
Degenerated blastocysts (%)		14		11		12		32
Data are presented as mean ± SE from three independent experiments.								

*Study II.* General health and fertility parameters in F0 dams. Daily exposure to dietary DEHP did not negatively affect the health of F0 dams during pregnancy and lactation. The abortion rate was 100% in the 500-mg DEHP dose group, but DEHP exposure did not significantly alter the litter size in the other groups (Table 3).

**Table 3:** Reproductive outcome of F0 dams treated with DEHP: study II.

DEHP (mg)						
0	0.05	5	500
Dams with positive plug (n)		15		14		13		14
Pregnant dams (n)		12		13		12		10
Delivery (n)		12		13		12		0
Abortion (%)		0		0		0		100
Litter size (mean ± SE)		5.24 ± 1.8		5.33 ± 1.9		4.83 ± 1.5		—
Data are presented as mean ± SE from two independent experiments.								

**Figure 5:**
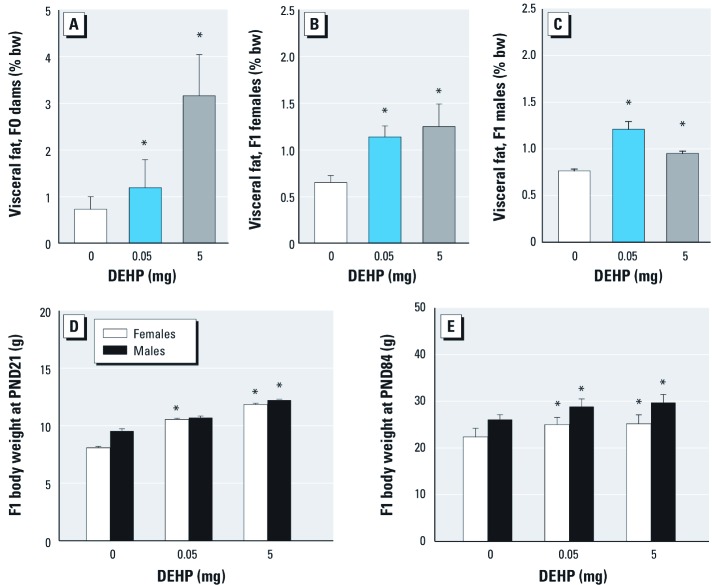
Effects of DEHP exposure on visceral fat and body weight: study II. Visceral fat by percentage of body weight in F0 dams after delivery and weaning (*A*; *n *= 15 ± 3 dams/group) and in F1 females (*B*) and males (*C*) at PND84 (*n *= 12 females/group or 12 males/group). Body weight of male and female F1 offspring at PND21 (*D*; *n *= 12 litters/group), and at PND84 (*E*; *n *= 12 females/group or 12 males/group). Results are mean ± SE of at least two independent experiments. *p < 0.05 compared with controls.

Visceral fat in F0 dams and F1 offspring (PND84). Visceral fat was increased in F0 dams (Figure 5A) and F1 females (Figure 5B), but the increase was not strictly monotonic with dose in F1 males (Figure 5C).

Body weight of F1 offspring at PND21 and PND84. The body weights of DEHP-exposed F1 female mice and F1 male mice in the 5-mg DEHP group were significantly higher than those of controls at PND21 (Figure 5D); however, body weights of F1 males in the 0.05-mg DEHP group were not significantly different from controls. The weight difference persisted until sacrifice at PND 84 (Figure 5E).

Average number of preimplantation F2 embryos. DEHP exposure of F1 mice was associated with a nonsignificant increase in the proportions of degenerated F2 embryos at 4 dpc in the 0.05 mg DEHP (28%) and 5 mg DEHP (29%) groups. We observed no significant differences in the average number of F2 embryos per mouse among all treatment groups (Table 4).

**Table 4:** Pregnancy rate and embryo survival in superovulated F1 females: study II.

DEHP (mg)				
0	0.05	5
Total dams (n)		15		15		15
Pregnant dams (n)		12		13		12
Flushed embryos/dam (mean ± SE)		10 ± 9.0		10 ± 5.0		7 ± 6.6
Degenerated blastocysts (%)		8		28		29
Data are presented as mean ± SE from two independent experiments.						

## Discussion

In study I, we observed increased visceral fat storage in DEHP-exposed F0 mice. Even the lowest DEHP dose (0.05 mg), which is comparable to the tolerable daily intake in humans issued by the WHO in 2003, led to a significant increase of visceral fat (WHO 2003). Dietary DEHP exposure induced an increase in food intake and an increase in body weight in all DEHP-exposure groups. Concurrent with the increase in food intake during the exposure period, weight gain rose significantly in all three experiments performed (Figure 1B); however, body weight increases were significantly different among the experiments (Figure 1C). This is supported by a previous study in which Hatch et al. (2008) reported a positive association between urinary phthalate metabolites and body mass index and waist circumference in women.

We found a significant (5.75-fold) increase in circulating plasma leptin concentration in the 500-mg DEHP group and a significant increase in leptin mRNA levels in adipose tissue in all DEHP-exposed F0 female mice compared with controls (study I). The concentration of leptin in plasma is an indicator of the amount of adipose tissue in mice (Frederich et al. 1995). The increased amount of visceral fat is consistent with higher leptin levels observed in DEHP-exposed mice. Elevated leptin levels, mediated by a central regulation in the hypothalamus via direct leptin receptor binding, normally reduce food consumption and body weight (Campfield et al. 1995). Although leptin levels were measured only at the end of the 8-week exposure period, we did not observe an association between leptin levels and food intake or body weight; this suggests an effect of DEHP on this negative feedback loop.

DEHP exposure was associated with a significant reduction of adiponectin mRNA expression in adipose tissue in all F0 DEHP-exposure groups. Consistent with this result is the observed adipocyte hypertrophy, which is in good agreement with the increase in leptin expression and the reduction in adiponectin expression. Adiponectin, predominantly expressed in adipocytes, increases glucose utilization and fatty acid oxidation in muscles and enhances insulin sensitivity in the liver (Kadowaki et al. 2006). Low plasma adiponectin levels are associated with an increased risk of metabolic syndrome and insulin resistance (Trujillo and Scherer 2005). *FABP4* mRNA expression in F0 females was increased in the 0.05-mg DEHP exposure group (study I). FABP4 is a specific carrier protein for fatty acids in adipocytes; elevated FABP4 serum levels have been associated with obesity and the metabolic syndrome in human studies (Terra et al. 2011; Xu et al. 2006). Our findings in study 1 suggest a potential role of FABP4 in effects of DEHP exposure on fat cell metabolism.

In addition to evidence of direct effects of DEHP in exposed F0 dams, we observed similar metabolic changes in F1 offspring after *in utero* and lactational exposure. At weaning, body weight of exposed F1 offspring in the 5-mg DEHP group was significantly increased in both sexes. Weight gain ranged between 22% in males and 33% in females (both in the 5-mg DEHP group). After weaning, F1 pups received a standard chow without DEHP. After 9 weeks without DEHP (PND84), body weights of DEHP-exposed F1 animals of both sexes were still significantly elevated. Moreover, fat storage in F1 females was significantly increased. DEHP is rapidly metabolized and excreted in urine and feces, and DEHP and its metabolites are known to cross the placenta and reach the fetus (Adibi et al. 2008; Wittassek et al. 2009). Results of our study II demonstrate a prolonged influence of *in utero* and lactational exposure to DEHP on body weight and adipose tissue formation from early infancy throughout adulthood.

In contrast to the effects of DEHP on food intake, body weight, and visceral fat tissue in C3H/N mice and their offspring observed here, studies in rats and mice that investigated reproductive effects of DEHP (Kluwe et al. 1982; Lin et al. 2011; Marsman 1995) reported a reduction in body weight. Strain differences due to gene mutations or enzyme inactivation accounting for differences in DEHP metabolism (Feige et al. 2010) may be a plausible explanation. In addition, recent cell culture studies on 3T3-L1 preadipocytes showed a promotion of adipogenesis by mono(2-ethylhexyl) phthalate (Feige et al. 2007) and dicyclohexyl phthalate (Sargis et al. 2009). Future studies seeking to determine whether the observed effects of DEHP were a consequence of increased food intake or metabolic effects independent of food intake should be pair-feeding transgenerational studies.

Lamb et al. (1987) studied fertility effects of DEHP in mice (both sexes) and found that DEHP caused dose-dependent decreases in fertility. Grande et al. (2006) reported that *in utero* and lactational exposure to DEHP (135 and 405 mg/kg/day) delayed the onset of puberty in female rat offspring. In a study by Takai et al. (2009), female rats exposed to a high dose of DEHP (3,000 mg/kg/day) had irregular estrous cycles and a slight decline in pregnancy rate. In our study I, the total number of F1 embryos per mouse was not altered by maternal DEHP exposure. However, in the 500-mg group, 32% of the blastocysts were degenerated, compared with 14% in controls (*p* = 0.0584). When we examined reproductive outcomes of DEHP-exposed F1 females (study II), we found that the total number of F2 embryos (exposed to DEHP only as germ cells) was not impaired. However, in the 0.05- and 5-mg DEHP groups, 28% and 29%, respectively, of the blastocysts were degenerated, compared with 8% of controls (*p* > 0.1299). Otherwise, exposure to DEHP did not alter the litter size in study II. In a study in rats, Takai et al. (2009) reported that a 2-fold higher DEHP treatment (1,000 mg/kg bw/day) over a period of 4 weeks did not disturb female fertility or early embryo development. Only the highest DEHP dose used in that study (3,000 mg/kg bw/day) affected pregnancy outcome.

## Conclusion

Dietary exposure to environmentally relevant concentrations of DEHP led to increases in food intake, body weight, and visceral fat tissue in female C3H/N mice (F0). Effects of DEHP were also observed in offspring of these females (offspring were exposed only *in utero* and during lactation; F1). These data suggest a predisposition for adiposity later in life in DEHP-exposed F1 mice. Doses of DEHP higher than those likely to be experienced in the environment interfered with the normal outcome of pregnancy and impaired early embryo development in C3H/N mice.
